# Supplementary material for: Bilateral trade potential analysis of the Lanzhou-Kathmandu South Asian rail-road freight trains linking China and Nepal: A stochastic frontier gravity model approach
Source: PLoS One. 2024 Jan 2;19(1):e0285325. doi: 10.1371/journal.pone.0285325 (PMC10760837; doi:10.1371/journal.pone.0285325)
Supplement: Attachment — Submitted filename: Response to Reviewers.docx [file pone.0285325.s001.docx]

Additional Editor Comments:

Dear author I have reviewed your manuscript where you focused the stochastic frontier gravity model is applied to analyze the trade potential between China and Nepal and the prospects of Lanzhou-Kathmandu South

Asian rail-road freight trains (LKSARFT), I invite you to make the improvement according reviewers' observations. Regarding to the observation reviewer in the point 4 I would like to suggest some references that you may be to change or add. In the first you can find stochastic Frontier models, and the second is a application of stochastic frontier model.

[1] Dios-Palomares et al. (2015) Analysis of the efficiency of farming systems in Latin America and the Caribbean considering environmental issues Revista Cientifica de la Facultad de Ciencias Veterinarias de la Universidad del Zulia, 2015, 25(1), pp. 43–50 (In Scupus)

[2] Zuniga-Gonzalez, C.A (2011). Technical efficienc stochastic y of organic fertilizer in small farms of Nicaragua: 1998-2005. AFRICAN JOURNAL OF BUSINESS MANAGEMENT Volume5 Issue3 Page967-973. https://www.webofscience.com/wos/woscc/full-record/WOS:000290682800034 (In WOS)

**Author’s respond**: We have added more descriptions about previous works in the literature review.

The SFGM is the Integration of Gravity Model and Stochastic Frontier Production Function Model which was formally introduced by [26] to address the inherent bias of the conventional gravity model of trade and to estimate potential trade flows. [53] applied frontier production function analysis to small farms in Nicaragua during 1998-2005, the results showed an acceptable average of technical efficiency which the makers of public policy in Nicaragua a must consider for the future. [40] focused on examining Philippines’s exports efficiency and potential based on trading partner’s characteristics using SFGM, unlike the usual measure of gravity model using OLS that measure potential from the mean. [12] provided a survey about environmental efficiency measure issue. the paper deals with different ways for including environmental variables, which offered several perspectives for measuring efficiency with frontier methods. [45] estimated the potential and efficiency of trade flows between China and countries along the Maritime Silk Road from 2005 to 2013 by using the SFGM. Furthermore, [17] defined the extent of the Belt and Road in terms of geographical boundaries, justified the application of the SFGM to the foreign direct investment (FDI) analysis, and constructed a frontier regression model to assess the China’s outward FDI efficiency.

[12] Dios-Palomares, R., Alcaide-López-de-Pablo, D., 2015. Analysis of the Efficiency of Farming Systems in Latin America and the Caribbean Considering Environmental Issues. [Revista Científica de Veterinaria](https://www.researchgate.net/journal/Revista-Cientifica-de-Veterinaria-0798-2259) 25(1), 43–50.

[53] Zuniga-Gonzalez, C.A., 2011. Technical efficiency of organic fertilizer in small farms of Nicaragua: 1998-2005. African Journal of Business Management 5(3), 967-973.

The data and information of this manuscript is relevant for publication in this journal, but you need to make the improvement by reviewers indicated.

Reviewers' comments:

Reviewer's Responses to Questions

Comments to the Author

1. Is the manuscript technically sound, and do the data support the conclusions?

The manuscript must describe a technically sound piece of scientific research with data that supports the conclusions. Experiments must have been conducted rigorously, with appropriate controls, replication, and sample sizes. The conclusions must be drawn appropriately based on the data presented.

Reviewer #1: Yes

Reviewer #2: Partly

2. Has the statistical analysis been performed appropriately and rigorously?

Reviewer #1: Yes

Reviewer #2: Yes

3. Have the authors made all data underlying the findings in their manuscript fully available?

The PLOS Data policy requires authors to make all data underlying the findings described in their manuscript fully available without restriction, with rare exception (please refer to the Data Availability Statement in the manuscript PDF file). The data should be provided as part of the manuscript or its supporting information, or deposited to a public repository. For example, in addition to summary statistics, the data points behind means, medians and variance measures should be available. If there are restrictions on publicly sharing data—e.g. participant privacy or use of data from a third party—those must be specified.

Reviewer #1: Yes

Reviewer #2: Yes

4. Is the manuscript presented in an intelligible fashion and written in standard English?

PLOS ONE does not copyedit accepted manuscripts, so the language in submitted articles must be clear, correct, and unambiguous. Any typographical or grammatical errors should be corrected at revision, so please note any specific errors here.

Reviewer #1: Yes

Reviewer #2: Yes

5. Review Comments to the Author

Please use the space provided to explain your answers to the questions above. You may also include additional comments for the author, including concerns about dual publication, research ethics, or publication ethics. (Please upload your review as an attachment if it exceeds 20,000 characters)

Reviewer #1: This paper is written well and explained clearly the Bilateral trade potential analysis of a rail-road freight train linking developing countries: A Stochastic Frontier Gravity Model approach. However, I have few comments that would help improve the quality of the paper.

1. It would be better if the author reports the P-value of Table 4 or indicate the statistical significance level using appropriate indicator.

**Author’s respond**: We have conducted statistical analysis on the P-value. Please see the additional content in Table 4.

Table 4

The basic regression analysis and model checking results based on SFGM

| **Independent variable** | **Exports model** | | | **Bilateral trade model** | | |
| --- | --- | --- | --- | --- | --- | --- |
|  | **Coefficient** | **T statistic** | **P value** | **Coefficient** | **T statistic** | **P value** |
| Constant term | 8.1740 | 5.7900 | 0.0000 | 24.5357 | 24.8255 | 0.0000 |
| $\mathrm{lnPGDP}_{i}$ | 0.2052 | 3.6128 | 0.0026 | -0.0711 | -0.6141 | 0.4635 |
| $\mathrm{lnPGDP}_{j}$ | 2.0500 | 13.0621 | 0.0000 | 0.1449 | 0.6506 | 0.4356 |
| $\mathrm{lnP}_{i}$ | 7.2513 | 2.4249 | 0.0039 | 0.0329 | 0.0347 | 0.9268 |
| $\mathrm{lnP}_{j}$ | -2.2414 | -15.9151 | 0.0000 | -2.0240 | -7.3138 | 0.0000 |
| $\ln\text{D}_{ij}$ | -3.0095 | -4.6072 | 0.0022 | -0.0596 | -0.6811 | 0.0001 |
| $\text{B}_{ij}$ | 0.5208 | 0.8756 | 0.4852 | 0.2101 | 0.2490 | 0.5486 |
| $\text{LAN}_{ij}$ | -0.1630 | -0.1650 | 0.8365 | -1.7590 | -10.3410 | 0.0000 |
| $\text{LANG}_{ij}$ | 5.0837 | 0.9458 | 0.3256 | 0.0127 | 0.0128 | 0.9325 |
| $\text{FTA}_{ij}$ | 1.2898 | 1.5898 | 0.1625 | 0.1515 | 0.1528 | 0.8735 |
| $\delta^{2}$ | 2.2449 | 6.8936 | 0.0001 | 21.3774 | 21.8081 | 0.0000 |
| $\gamma$ | 0.9999 | 432.9989 | 0.0000 | 0.8433 | 17302.4610 | 0.0000 |
| $\mu$ | -2.5120 | -4.8310 | 0.0019 | 0.0850 | 0.0850 | 0.9023 |
| $\eta$ | -0.0040 | -0.1850 | 0.8269 | 0.0570 | 0.0670 | 0.9125 |
| Log likelihood | -173.2440 | —— | —— | -262.7431 | —— | —— |
| LR Statistics | 252.3511 | —— | —— | 80.2145 | —— | —— |

2. The authors need to discuss more policy implications of there study.

**Author’s respond**: We have prescribed the policy implications based on the study results in a separate paragraph after the study conclusions. Please see the Penultimate paragraph.

In line with these findings, we give policy directions to boost bilateral trade efficiency and tap the potential of bilateral trade between the two countries: (i) Export goods would be stimulated by formulating and implementing macro-economic policies aimed at increasing the economic size of China. (ii) The establishment of a robust and comprehensive integrated process for foreign exports would facilitate the process of foreign exports, consequently reducing China's export trade impediments with Nepal. (iii) In order to mitigate transportation costs, China should establish and implement tax incentive policies for cross-border trade in encouraged sectors. (iv) Both countries should promote the teaching of each other's languages in their respective education systems to enhance communication and cooperation, and facilitate cross-cultural understanding, thereby promoting trade development between the two nations. (iv) To rectify China's trade surplus, it is imperative for the nation to calibrate its fiscal policies such that there is a surge in demand, thereby inducing an increase in import volume.

3. The authors reveal within the manuscript that the data set consist Bangladesh, Pakistan, India, Bhutan, Sri Lanka, Nepal Afghanistan, & Maldives. But the authors give much emphasis on Nepal only. I understand this, the discussion also must mention other countries involve.

**Author’s respond**: We have added more descriptions about discussions pertaining to other countries in section 3 and section 4. Please see the red part.

Table 2

The needed initial data of the eight South Asian countries (from 2001 to 2018)

|  | Year | $\mathrm{EAI}_{ijt}$  (Billion USD) | $\text{EXP}_{ijt}$  (Billion USD) | $\mathrm{PGDP}_{it}$  (USD) | $\mathrm{PGDP}_{jt}$  (USD) | $\mathrm{P}_{it}$  (Billion) | $\mathrm{P}_{jt}$  (Billion) |
| --- | --- | --- | --- | --- | --- | --- | --- |
| $i$: China  $j$: Nepal | 2001 | 1.532 | 1.486 | 1041.64 | 240.47 | 12.72 | 0.250 |
|  | 2002 | 1.103 | 1.051 | 1135.45 | 236.71 | 12.80 | 0.256 |
|  | 2003 | 1.273 | 1.220 | 1273.64 | 242.14 | 12.88 | 0.261 |
|  | 2004 | 1.715 | 1.632 | 1490.38 | 272.25 | 12.96 | 0.267 |
|  | 2005 | 1.964 | 1.879 | 1731.13 | 298.01 | 13.04 | 0.273 |
|  | 2006 | 2.680 | 2.598 | 2069.34 | 326.04 | 13.11 | 0.287 |
|  | 2007 | 4.000 | 3.860 | 2651.26 | 362.22 | 13.18 | 0.284 |
|  | 2008 | 3.810 | 3.750 | 3413.59 | 434.96 | 13.25 | 0.290 |
|  | 2009 | 4.140 | 4.090 | 3748.93 | 438.29 | 13.31 | 0.294 |
|  | 2010 | 7.437 | 7.322 | 4432.96 | 534.52 | 13.38 | 0.300 |
|  | 2011 | 11.950 | 11.810 | 5444.79 | 619.45 | 13.44 | 0.305 |
|  | 2012 | 19.980 | 19.680 | 6188.19 | 706.65 | 13.51 | 0.275 |
|  | 2013 | 22.540 | 22.100 | 6807.43 | 694.10 | 13.57 | 0.278 |
|  | 2014 | 23.300 | 22.830 | 7593.88 | 696.94 | 13.64 | 0.282 |
|  | 2015 | 28.660 | 27.890 | 7924.65 | 732.30 | 13.71 | 0.285 |
|  | 2016 | 30.245 | 28.900 | 8000.34 | 756.44 | 13.72 | 0.299 |
|  | 2017 | 32.335 | 31.008 | 8123.22 | 788.00 | 13.98 | 0.310 |
|  | 2018 | 35.667 | 33.450 | 8256.77 | 812.22 | 14.31 | 0.334 |
| $i$: China  $j$: India | 2001 | 35.960 | 18.960 | 1041.64 | 459.58 | 12.72 | 10.71 |
|  | 2002 | 49.460 | 26.710 | 1135.45 | 480.21 | 12.80 | 10.89 |
|  | 2003 | 75.950 | 33.430 | 1273.64 | 558.44 | 12.88 | 11.06 |
|  | 2004 | 136.040 | 59.360 | 1490.38 | 642.56 | 12.96 | 11.23 |
|  | 2005 | 187.030 | 89.340 | 1731.13 | 731.74 | 13.04 | 11.40 |
|  | 2006 | 248.600 | 145.810 | 2069.34 | 820.30 | 13.11 | 11.57 |
|  | 2007 | 386.500 | 240.110 | 2651.26 | 1055.14 | 13.18 | 11.74 |
|  | 2008 | 518.400 | 315.850 | 3413.59 | 1027.91 | 13.25 | 11.91 |
|  | 2009 | 433.830 | 296.560 | 3748.93 | 1126.95 | 13.31 | 12.08 |
|  | 2010 | 617.610 | 409.150 | 4432.96 | 1375.39 | 13.38 | 12.25 |
|  | 2011 | 739.080 | 505.370 | 5444.79 | 1488.52 | 13.44 | 12.41 |
|  | 2012 | 687.900 | 539.400 | 6188.19 | 1489.24 | 13.51 | 12.37 |
|  | 2013 | 654.700 | 484.400 | 6807.43 | 1498.87 | 13.57 | 12.52 |
|  | 2014 | 706.050 | 542.260 | 7593.88 | 1595.70 | 13.64 | 12.95 |
|  | 2015 | 716.200 | 582.400 | 7924.65 | 1581.59 | 13.71 | 13.11 |
|  | 2016 | 755.400 | 678.900 | 8000.34 | 1599.00 | 13.72 | 13.22 |
|  | 2017 | 809.998 | 599.450 | 8123.22 | 1601.22 | 13.98 | 13.56 |
|  | 2018 | 825.445 | 601.220 | 8256.77 | 1598.88 | 14.31 | 13.89 |
| $i$: China  $j$: Pakistan | 2001 | 13.970 | 8.150 | 1041.64 | 490.04 | 12.72 | 1.48 |
|  | 2002 | 17.995 | 12.420 | 1135.45 | 480.74 | 12.80 | 1.50 |
|  | 2003 | 24.300 | 18.550 | 1273.64 | 543.59 | 12.88 | 1.53 |
|  | 2004 | 30.610 | 24.660 | 1490.38 | 628.63 | 12.96 | 1.56 |
|  | 2005 | 42.610 | 34.280 | 1731.13 | 690.85 | 13.04 | 1.59 |
|  | 2006 | 52.460 | 42.390 | 2069.34 | 789.41 | 13.11 | 1.62 |
|  | 2007 | 68.930 | 57.890 | 2651.26 | 870.63 | 13.18 | 1.64 |
|  | 2008 | 70.570 | 60.510 | 3413.59 | 978.80 | 13.25 | 1.67 |
|  | 2009 | 67.880 | 55.280 | 3748.93 | 949.12 | 13.31 | 1.70 |
|  | 2010 | 86.680 | 69.370 | 4432.96 | 1018.87 | 13.38 | 1.74 |
|  | 2011 | 105.570 | 84.390 | 5444.79 | 1194.33 | 13.44 | 1.77 |
|  | 2012 | 124.130 | 92.750 | 6188.19 | 1290.36 | 13.51 | 1.79 |
|  | 2013 | 142.150 | 110.190 | 6807.43 | 1299.12 | 13.57 | 1.82 |
|  | 2014 | 160.060 | 132.480 | 7593.88 | 1334.15 | 13.64 | 1.85 |
|  | 2015 | 189.300 | 164.500 | 7924.65 | 1428.99 | 13.71 | 1.89 |
|  | 2016 | 219.000 | 189.000 | 8000.34 | 1521.80 | 13.72 | 1.99 |
|  | 2017 | 225.340 | 210.990 | 8123.22 | 1788.90 | 13.98 | 1.97 |
|  | 2018 | 279.900 | 235.790 | 8256.77 | 1899.90 | 14.31 | 1.89 |
| $i$: China  $j$: Bangladesh | 2001 | 9.720 | 9.550 | 1041.64 | 356.12 | 12.72 | 1.32 |
|  | 2002 | 11.000 | 10.680 | 1135.45 | 354.30 | 12.80 | 1.34 |
|  | 2003 | 13.700 | 13.360 | 1273.64 | 380.28 | 12.88 | 1.37 |
|  | 2004 | 19.660 | 19.090 | 1490.38 | 407.99 | 12.96 | 1.39 |
|  | 2005 | 24.800 | 24.020 | 1731.13 | 428.75 | 13.04 | 1.41 |
|  | 2006 | 31.890 | 30.900 | 2069.34 | 434.84 | 13.11 | 1.42 |
|  | 2007 | 34.590 | 33.450 | 2651.26 | 475.25 | 13.18 | 1.44 |
|  | 2008 | 46.850 | 45.540 | 3413.59 | 546.85 | 13.25 | 1.45 |
|  | 2009 | 45.820 | 44.410 | 3748.93 | 607.76 | 13.31 | 1.47 |
|  | 2010 | 70.570 | 67.890 | 4432.96 | 674.93 | 13.38 | 1.49 |
|  | 2011 | 82.600 | 78.100 | 5444.79 | 735.00 | 13.44 | 1.50 |
|  | 2012 | 84.500 | 79.700 | 6188.19 | 747.34 | 13.51 | 1.55 |
|  | 2013 | 103.070 | 97.050 | 6807.43 | 829.25 | 13.57 | 1.57 |
|  | 2014 | 125.430 | 117.820 | 7593.88 | 1092.67 | 13.64 | 1.59 |
|  | 2015 | 147.070 | 139.010 | 7924.65 | 1211.70 | 13.71 | 1.61 |
|  | 2016 | 155.230 | 141.909 | 8000.34 | 1467.88 | 13.72 | 1.67 |
|  | 2017 | 159.990 | 145.078 | 8123.22 | 1541.11 | 13.98 | 1.78 |
|  | 2018 | 162.786 | 146.099 | 8256.77 | 1578.09 | 14.31 | 1.78 |
| $i$: China  $j$: Afghanistan | 2001 | 0.174 | 0.172 | 1041.64 | 92.21 | 12.72 | 0.267 |
|  | 2002 | 0.199 | 0.199 | 1135.45 | 157.98 | 12.80 | 0.274 |
|  | 2003 | 0.271 | 0.265 | 1273.64 | 168.68 | 12.88 | 0.282 |
|  | 2004 | 0.579 | 0.569 | 1490.38 | 196.23 | 12.96 | 0.290 |
|  | 2005 | 0.527 | 0.512 | 1731.13 | 227.88 | 13.04 | 0.299 |
|  | 2006 | 1.006 | 1.004 | 2069.34 | 251.11 | 13.11 | 0.307 |
|  | 2007 | 1.718 | 1.694 | 2651.26 | 306.98 | 13.18 | 0.316 |
|  | 2008 | 1.543 | 1.516 | 3413.59 | 367.19 | 13.25 | 0.325 |
|  | 2009 | 2.148 | 2.135 | 3748.93 | 425.07 | 13.31 | 0.334 |
|  | 2010 | 1.789 | 1.752 | 4432.96 | 501.47 | 13.38 | 0.343 |
|  | 2011 | 2.344 | 2.300 | 5444.79 | 575.97 | 13.44 | 0.353 |
|  | 2012 | 4.692 | 4.640 | 6188.19 | 575.97 | 13.51 | 0.298 |
|  | 2013 | 3.378 | 3.282 | 6807.43 | 678.35 | 13.57 | 0.305 |
|  | 2014 | 4.109 | 3.393 | 7593.88 | 658.98 | 13.64 | 0.316 |
|  | 2015 | 3.760 | 3.640 | 7924.65 | 590.27 | 13.71 | 0.325 |
|  | 2016 | 3.980 | 3.780 | 8000.34 | 599.01 | 13.72 | 0.356 |
|  | 2017 | 4.009 | 3.889 | 8123.22 | 600.89 | 13.98 | 0.339 |
|  | 2018 | 4.145 | 3.990 | 8256.77 | 611.29 | 14.31 | 0.390 |
| $i$: China  $j$: Bhutan | 2001 | 0.0162 | 0.0160 | 1041.64 | 774.89 | 12.72 | 0.0588 |
|  | 2002 | 0.0064 | 0.0062 | 1135.45 | 837.05 | 12.80 | 0.0606 |
|  | 2003 | 0.0198 | 0.0197 | 1273.64 | 978.51 | 12.88 | 0.0624 |
|  | 2004 | 0.0052 | 0.0035 | 1490.38 | 1074.58 | 12.96 | 0.0642 |
|  | 2005 | 0.0047 | 0.0047 | 1731.13 | 1242.04 | 13.04 | 0.0659 |
|  | 2006 | 0.0016 | 0.0016 | 2069.34 | 1330.52 | 13.11 | 0.0674 |
|  | 2007 | 0.0539 | 0.0539 | 2651.26 | 1736.97 | 13.18 | 0.0688 |
|  | 2008 | 0.0846 | 0.0846 | 3413.59 | 1792.91 | 13.25 | 0.0701 |
|  | 2009 | 0.0417 | 0.0412 | 3748.93 | 1772.10 | 13.31 | 0.0713 |
|  | 2010 | 0.0160 | 0.0159 | 4432.96 | 2088.49 | 13.38 | 0.0725 |
|  | 2011 | 0.1746 | 0.1738 | 5444.79 | 2287.71 | 13.44 | 0.0738 |
|  | 2012 | 0.1562 | 0.1560 | 6188.19 | 2398.91 | 13.51 | 0.0741 |
|  | 2013 | 0.1741 | 0.1740 | 6807.43 | 2498.39 | 13.57 | 0.0753 |
|  | 2014 | 0.1122 | 0.1112 | 7593.88 | 2380.91 | 13.64 | 0.0765 |
|  | 2015 | 0.1531 | 0.1520 | 7924.65 | 2532.45 | 13.71 | 0.0774 |
|  | 2016 | 0.1677 | 0.1677 | 8000.34 | 2667.89 | 13.72 | 0.0788 |
|  | 2017 | 0.1998 | 0.1690 | 8123.22 | 2776.90 | 13.98 | 0.0791 |
|  | 2018 | 0.2319 | 0.1709 | 8256.77 | 2890.00 | 14.31 | 0.0801 |
| $i$: China  $j$: Sri Lanka | 2001 | 3.967 | 3.866 | 1041.64 | 837.70 | 12.72 | 0.187 |
|  | 2002 | 3.510 | 3.367 | 1135.45 | 903.90 | 12.80 | 0.189 |
|  | 2003 | 5.240 | 5.040 | 1273.64 | 984.81 | 12.88 | 0.191 |
|  | 2004 | 7.170 | 6.940 | 1490.38 | 1063.16 | 12.96 | 0.194 |
|  | 2005 | 9.760 | 9.390 | 1731.13 | 1242.40 | 13.04 | 0.196 |
|  | 2006 | 11.410 | 11.060 | 2069.34 | 1423.48 | 13.11 | 0.198 |
|  | 2007 | 14.320 | 13.840 | 2651.26 | 1614.41 | 13.18 | 0.200 |
|  | 2008 | 16.900 | 16.300 | 3413.59 | 2013.91 | 13.25 | 0.202 |
|  | 2009 | 16.390 | 15.680 | 3748.93 | 2057.11 | 13.31 | 0.204 |
|  | 2010 | 20.970 | 19.940 | 4432.96 | 2400.02 | 13.38 | 0.206 |
|  | 2011 | 31.410 | 29.880 | 5444.79 | 2835.41 | 13.44 | 0.208 |
|  | 2012 | 31.630 | 30.010 | 6188.19 | 2923.13 | 13.51 | 0.203 |
|  | 2013 | 36.190 | 34.360 | 6807.43 | 3279.89 | 13.57 | 0.204 |
|  | 2014 | 40.410 | 37.920 | 7593.88 | 3631.05 | 13.64 | 0.206 |
|  | 2015 | 40.300 | 37.300 | 7924.65 | 3926.17 | 13.71 | 0.209 |
|  | 2016 | 41.009 | 37.890 | 8000.34 | 4009.31 | 13.72 | 0.208 |
|  | 2017 | 41.890 | 37.990 | 8123.22 | 4109.90 | 13.98 | 0.210 |
|  | 2018 | 42.009 | 38.009 | 8256.77 | 4289.99 | 14.31 | 0.221 |
| $i$: China  $j$: Maldives | 2001 | 0.0220 | 0.0210 | 1041.64 | 2838.07 | 12.72 | 0.00277 |
|  | 2002 | 0.0298 | 0.0296 | 1135.45 | 2885.49 | 12.80 | 0.00282 |
|  | 2003 | 0.0335 | 0.0334 | 1273.64 | 3251.62 | 12.88 | 0.00286 |
|  | 2004 | 0.0809 | 0.0791 | 1490.38 | 3638.57 | 12.96 | 0.00291 |
|  | 2005 | 0.1696 | 0.1693 | 1731.13 | 3361.59 | 13.04 | 0.00295 |
|  | 2006 | 0.1600 | 0.1540 | 2069.34 | 4353.01 | 13.11 | 0.00299 |
|  | 2007 | 0.2500 | 0.2470 | 2651.26 | 5079.99 | 13.18 | 0.00303 |
|  | 2008 | 0.3290 | 0.3150 | 3413.59 | 6149.01 | 13.25 | 0.00307 |
|  | 2009 | 0.4080 | 0.4060 | 3748.93 | 6229.67 | 13.31 | 0.00311 |
|  | 2010 | 0.6350 | 0.6340 | 4432.96 | 6570.43 | 13.38 | 0.00315 |
|  | 2011 | 0.9730 | 0.9710 | 5444.79 | 6405.05 | 13.44 | 0.00320 |
|  | 2012 | 0.7660 | 0.7640 | 6188.19 | 6566.65 | 13.51 | 0.00338 |
|  | 2013 | 0.9780 | 0.9740 | 6807.43 | 6665.77 | 13.57 | 0.00345 |
|  | 2014 | 1.0430 | 1.0390 | 7593.88 | 8483.81 | 13.64 | 0.00357 |
|  | 2015 | 1.0920 | 1.0400 | 7924.65 | 7681.08 | 13.71 | 0.00409 |
|  | 2016 | 1.1098 | 1.0448 | 8000.34 | 7767.89 | 13.72 | 0.00412 |
|  | 2017 | 1.0989 | 1.0489 | 8123.22 | 7890.90 | 13.98 | 0.00467 |
|  | 2018 | 1.0998 | 1.0509 | 8256.77 | 7998.12 | 14.31 | 0.00489 |

Table 3

Other initial data of the eight South Asian countries

|  | $\text{D}_{ij}$(kilometer) |  |  | $\text{LANG}_{ij}$ | $\text{FTA}_{ij}$ |
| --- | --- | --- | --- | --- | --- |
| $i$: China,$j$: Nepal | 1976.1 | 1 | 0 | 1 | 0 |
| $i$: China,$j$: India | 2620.7 | 1 | 0 | 1 | 0 |
| $i$: China,$j$: Pakistan | 2802.8 | 1 | 0 | 1 | 1 |
| $i$: China,$j$: Bangladesh | 1874.6 | 0 | 0 | 1 | 0 |
| $i$: China,$j$: Afghanistan | 3129.0 | 1 | 0 | 1 | 0 |
| $i$: China,$j$: Bhutan | 1644.0 | 1 | 0 | 1 | 0 |
| $i$: China,$j$: Sri Lanka | 4043.0 | 0 | 0 | 1 | 0 |
| $i$: China,$j$: Maldives | 4711.0 | 0 | 0 | 1 | 0 |

In this paper we study the LKSARFT, so$\text{D}_{ij}$ are the straight line distances between Lanzhou to the capital city of other related countries: Lanzhou-Kathmandu, Lanzhou-New Delhi, Lanzhou-Islamabad, Lanzhou-Dhaka, Lanzhou-Kabul, Lanzhou-Thimphu, Lanzhou-Colombo, Lanzhou-Male.

The results presented in Table 2 demonstrate a steady increase in the bilateral trade volume between China and the eight South Asian countries, as well as China's export volume to these nations from 2001 to 2018. Notably, the bilateral trade volume and export amount between China and India was the highest among the eight countries. The rankings of bilateral trade volume and export amount between China and the South Asian countries from one to eight are as follows: India, Pakistan, Bangladesh, Sri Lanka, Nepal, Afghanistan, Maldives, and Bhutan. Furthermore, the ranking of China's export amount to the eight South Asian countries from one to eight is also as follows: India, Pakistan, Bangladesh, Sri Lanka, Nepal, Afghanistan, Maldives, and Bhutan. The subsequent discussion will primarily focus on Nepal.

4. Results and discussion

4.1. The results and analysis

First of all, we should test whether the Eq.(6) and Eq.(7) are suitable to assess the performance of bilateral potential trade among the countries mentioned above. The testing results were presented in Table 4. We refused the assumptions because all LR Statistics values were larger than critical value, which means the two model are suitable to describe the non-efficiency trade, and the non-efficiency trade changes when time changes.

Table 4

The model testing results

|  | $H_{0}$ | $\ln\left(H_{0}\right)$ | $\ln\left(H_{1}\right)$ | **LR statistics value** | **Critical value (1%)** | **Conclusion** |
| --- | --- | --- | --- | --- | --- | --- |
| Eq.(6) | $\gamma=\mu=\eta=0$ | -173.2444 | -299.4199 | 252.3511 | 11.34 | Refuse |
|  | $\eta=0$ | -299.4199 | -391.4562 | 183.8726 | 6.63 | Refuse |
| Eq.(7) | $\gamma=\mu=\eta=0$ | -302.8503 | -342.9575 | 80.2145 | 9.21 | Refuse |
|  | $\eta=0$ | -262.7431 | -272.3425 | 19.1988 | 6.63 | Refuse |

We used Frontier 4.1 software to regression analyze the initial data mentioned in Table 2 and 3. The basic regression analysis and the model checking results were presented in Table 5. The trade efficiency analysis results between China and Nepal were presented in Table 6, including exports efficiency (EE) and bilateral trade efficiency (BTE), as well as the rankings of EE and BTE among the eight South Asian countries. The trade potential measurement results between China and Nepal were showed in Table 7.

Table 5

The basic regression analysis and model checking results based on SFGM

| **Independent variable** | **Exports model** | | | **Bilateral trade model** | | |
| --- | --- | --- | --- | --- | --- | --- |
|  | **Coefficient** | **T statistic** | **P value** | **Coefficient** | **T statistic** | **P value** |
| Constant term | 8.1740 | 5.7900 | 0.0000 | 24.5357 | 24.8255 | 0.0000 |
| $\mathrm{lnPGDP}_{i}$ | 0.2052 | 3.6128 | 0.0026 | -0.0711 | -0.6141 | 0.4635 |
| $\mathrm{lnPGDP}_{j}$ | 2.0500 | 13.0621 | 0.0000 | 0.1449 | 0.6506 | 0.4356 |
| $\mathrm{lnP}_{i}$ | 7.2513 | 2.4249 | 0.0039 | 0.0329 | 0.0347 | 0.9268 |
| $\mathrm{lnP}_{j}$ | -2.2414 | -15.9151 | 0.0000 | -2.0240 | -7.3138 | 0.0000 |
| $\ln\text{D}_{ij}$ | -3.0095 | -4.6072 | 0.0022 | -0.0596 | -0.6811 | 0.0001 |
| $\text{B}_{ij}$ | 0.5208 | 0.8756 | 0.4852 | 0.2101 | 0.2490 | 0.5486 |
| $\text{LAN}_{ij}$ | -0.1630 | -0.1650 | 0.8365 | -1.7590 | -10.3410 | 0.0000 |
| $\text{LANG}_{ij}$ | 5.0837 | 0.9458 | 0.3256 | 0.0127 | 0.0128 | 0.9325 |
| $\text{FTA}_{ij}$ | 1.2898 | 1.5898 | 0.1625 | 0.1515 | 0.1528 | 0.8735 |
| $\delta^{2}$ | 2.2449 | 6.8936 | 0.0001 | 21.3774 | 21.8081 | 0.0000 |
| $\gamma$ | 0.9999 | 432.9989 | 0.0000 | 0.8433 | 17302.4610 | 0.0000 |
| $\mu$ | -2.5120 | -4.8310 | 0.0019 | 0.0850 | 0.0850 | 0.9023 |
| $\eta$ | -0.0040 | -0.1850 | 0.8269 | 0.0570 | 0.0670 | 0.9125 |
| Log likelihood | -173.2440 | —— | —— | -262.7431 | —— | —— |
| LR Statistics | 252.3511 | —— | —— | 80.2145 | —— | —— |

After analyzing the Table 5, we could obtain the following conclusions:

(i) For both the exports model and bilateral trade model,$\gamma\neq0$ means non-efficiency trade’s influence on trade efficiency in two models are 84.33% and 99.99%, respectively. The exports model’s$\gamma$ value is larger than the bilateral trade model, which means exports trade resistance from China to Nepal is larger than the bilateral trade resistance.
(ii) For both the exports model and bilateral trade model,$\mu\neq0$ shows that the non-efficiency factors existed in the trade processes, the SFGM is suitable to describe the trade between China and Nepal.
(iii) For the exports model and bilateral trade model,$\eta\neq0$ shows that the time-varying SFGMs are suitable to describe the trade between China and Nepal. For the bilateral trade model,$\eta=0.057$ shows the bilateral non-efficiency factor decreasing at a rate of 0.057 with time increasing, and the bilateral trade increasing at a rate of 0.057 with time increasing; For the exports model,$\eta=-0.004$ means the exports non-efficiency factor increasing at a rate of 0.004 with time increasing, exports trade decreasing at a rate of 0.057 with time increasing.
(iv) For both the exports model and bilateral trade model, elasticity of per capita GDP in China is less than the value of the other seven trade countries, shows that the economic size of the trade countries has a great influence on China’s exports and bilateral trade. It is necessary to evaluate the seven trade countries’ economic development in the future if China wants more trades among the seven countries.
(v) The coefficients of the trade countries’ population are negative in the two models, indicates that if the trade countries have small number of population, they have smaller domestic markets, and they have smaller imports from China as well as bilateral trades. On the contrary, the coefficients of China’s population are negative in the two models; shows that the larger population constitutes a larger domestic market and larger imports demand form the trade countries. We thought that China’s labor force advantages have changed into the trade advantages.
(vi) The coefficients of the distance between two trade countries are negative in the two models. When the distance between two trading countries increased 1%, the exports trade value decreased 3.009%, and the bilateral trade value decreased 0.059%. Longer distance means larger transportation costs, which limits the development of trades.
(vii) The coefficients of and are negative in the exports model. Most of the countries sharing a common border with China are located in inner Asia-Europe Continent, the land transportation corridors development between two bordering countries are limited by the geographical environment, e.g., the mountains. Compared with the landlocked countries, China has both territorial land and territorial sea, the shipping provides a greater convenience for China’s exports trade.
(viii) The coefficients of are positive in the two models, English is the commonly used language among China and other counties while trading, it is helpful to improve the trade development if two trading countries share one common language.
(ix) The coefficients of$\text{FTA}_{ij}$ are positive in the two models, which means the free trade agreement is critical to reduce bilateral trade barriers and trade non-efficiency factors.

Table 6

Exports efficiency (EE) and bilateral trade efficiency (BTE) results between China and Nepal based on SFGM

| **Year** | **EE** | **EE Rank** | **BTE** | **BTE Rank** | **Year** | **EE** | **EE Rank** | **BTE** | **BTE Rank** |
| --- | --- | --- | --- | --- | --- | --- | --- | --- | --- |
| 2001 | 0.1324 | 4 | 0.0025 | 5 | 2010 | 0.0863 | 4 | 0.0124 | 5 |
| 2002 | 0.0744 | 4 | 0.0020 | 5 | 2011 | 0.1313 | 4 | 0.0179 | 5 |
| 2003 | 0.1053 | 4 | 0.0021 | 5 | 2012 | 0.1280 | 4 | 0.0322 | 5 |
| 2004 | 0.0837 | 4 | 0.0031 | 5 | 2013 | 0.1850 | 4 | 0.0338 | 5 |
| 2005 | 0.1035 | 4 | 0.0032 | 5 | 2014 | 0.1514 | 4 | 0.0378 | 5 |
| 2006 | 0.0883 | 4 | 0.0047 | 5 | 2015 | 0.1986 | 4 | 0.0434 | 5 |
| 2007 | 0.1379 | 4 | 0.0063 | 5 | 2016 | 0.2088 | 4 | 0.0452 | 5 |
| 2008 | 0.0688 | 4 | 0.0065 | 5 | 2017 | 0.2134 | 4 | 0.0466 | 5 |
| 2009 | 0.0959 | 4 | 0.0064 | 5 | 2018 | 0.2451 | 4 | 0.0478 | 5 |

The results in Table 6 show that larger efficiency value, larger EE and BTE, and smaller trade potential. Otherwise, smaller efficiency value, smaller EE and BTE, means larger trade potential. The BTE between China and Nepal increases when time changes, the EE from China to Nepal remains constant changing during the 18 years. The changing range of BTE is 0.002-0.05; the changing range of EE from China to Nepal is over 0.1, larger than the BTE. The BTE and EE ranking among the eight South Asian countries are ranking fifth and fourth during the 18 years (BTE ranking from 1 to 8: India, Pakistan, Bangladesh, Sri Lanka, Nepal, Afghanistan, Maldives, Bhutan; EE ranking from 1 to 8: India, Bangladesh, Pakistan, Nepal, Afghanistan, Bhutan, Sri Lanka, Maldives). The increasing BTE shows that the trade between China and Nepal has improved a lot; the trade potential has a lot of room to improve. Larger EE means the smaller exports trade potential, increasingly saturated exports markets from China to Nepal, some measures must be applied to improve China’s trade surplus.

The ratio of actual trade volume (EXP and EAI) and efficiency (EE and BTE, presented in Table 6) represents the Exports Trade Potential (ETP) and Bilateral Trade Potential (BTP), respectively. We could obtain the Extended Exports Trade Potential (EETP) and Extended Bilateral Trade Potential (EBTP) by substituting the original data (presented in Table 1 and Table 2) into the estimated random frontier gravity equations. The difference between ETP and EETP (BEP and EBTP) is, ETP (BEP) is influenced by the economic data of other seven countries, and EETP (EBTP) is the economic indicator which reflects the pure economic trade potential between China and Nepal. We subtracted 100% from the ratio of extended trade potential (EETP and EBTP) and trade potential (ETP and BTP), the results presented the Improved Exports Trade Potential (IETP) and Improved Bilateral Trade Potential (IBTP), respectively. All results were presented in Table 7.

4. The literature reviewed are inadequate, a lot of recent related studies are exempted from the review studies such as: Abdullahi et. al. (2022). Examining the determinants and efficiency of China’s agricultural exports using a stochastic frontier gravity model. Abdullahi, Aluko & Huo. (2021). Determinants, efficiency and potential of agri-food exports from Nigeria to the EU: Evidence from the stochastic frontier gravity model. Abdullahi et al. (2021). Determinants and Potential of Agri-Food Trade Using the Stochastic Frontier Gravity Model: Empirical Evidence From Nigeria. Jiang & Zhang (2021). Trade Sustainability and Efficiency under the Belt and Road Initiative: A Stochastic Frontier Analysis of China’s Trade Potential at Industry Level.

**Author’s respond**: We have added more descriptions about previous works in the literature review.

[1] used an extended gravity model to examine the determinants, efficiency and potential of agri-food exports from Nigeria to the EU for the 1995-2019 period the study showed that Nigeria’s agri-food exports with the EU has a relatively large potential that has not been exploited. The stochastic frontier gravity model is applied on a dataset including 35 countries during 2009–2017 by [24], the results indicated that the trade resistance of China’s export to countries along the Belt and Road has increased over time, while there is still huge trade potential at various industries. [2] provided empirical insights on the determinants and potential of agri-food exports from Nigeria to 70 major trading countries between 1995 and 2019 by applying a Stochastic Frontier Analysis on a gravity model. [3] aimed to examine the key determinants and efficiency of China’s agricultural exports with its 114 importing countries by applying the Stochastic Frontier Analysis on an augmented gravity model for the period of 2000–2019.

[1] Abdullahi, N. M., Aluko, O. A., Huo, X., 2021. Determinants, efficiency and potential of agri-food exports from nigeria to the eu: evidence from the stochastic frontier gravity model. Agricultural Economics (AGRICECON). 67(8), 337-349.

[2] Abdullahi, N. M., Huo, X., Zhang, Q., Azeez, A. B., 2021. Determinants and potential of agri-food trade using the stochastic frontier gravity model: empirical evidence from nigeria. SAGE Open. 11(4), 1-12.

[3] Abdullahi, N. M., Zhang, Q., Shahriar, S., Huo, X., 2022. Examining the determinants and efficiency of China’s agricultural exports using a stochastic frontier gravity model. PLoS ONE. 17(9), 0274187.

[24] Jiang, W., Zhang, H., Lin, Y., 2021. Trade sustainability and efficiency under the belt and road initiative: a stochastic frontier analysis of China's trade potential at industry level. Emerging Markets Finance and Trade. 58(1), 1-13.

Reviewer #2: 1. Title: The manuscript title is general and it is recommended to make it specific.

**Author’s respond**: We have revised the title.

Bilateral trade potential analysis of the Lanzhou-Kathmandu South Asian rail-road freight trains linking China and Nepal: A Stochastic Frontier Gravity Model approach.

2. Abstract: This section has been descriptively written. It is suggested to include the findings of the study in quantitate form here. In addition, policy implications should be included concisely at the end of this section.

**Author’s respond**: We have added the findings of the study in quantitate and policy implications in Abstract.

In this paper, the stochastic frontier gravity model is applied to analyze the trade potential between China and Nepal and the prospects of Lanzhou-Kathmandu South Asian rail-road freight trains (LKSARFT). Based on the statistical data, we test the Exports Efficiency (EE), Bilateral Trade Efficiency (BTE), Exports Trade Potential (ETP), Bilateral Trade Potential (BTP), Extended Exports Trade Potential (EETP), Extended Bilateral Trade Potential (EBTP), Improved Exports Trade Potential (IETP) and Improved Bilateral Trade Potential (IBTP) between China and Nepal, the following analysis results can be found: for the bilateral trade model, the bilateral non-efficiency factor decreasing at a rate of 0.057 with time increasing, bilateral trade increasing at a rate of 0.057 with time increasing. For the exports model, the exports non-efficiency factor increasing at a rate of 0.004 with time increasing, exports trade decreasing at a rate of 0.057 with time increasing. The BTE between China and Nepal increases when time changes, the EE from China to Nepal remains constant changing during the 18 years. The changing range of BTE is 0.002-0.05; the changing range of EE from China to Nepal is over 0.1, larger than the BTE. The BTE and EE ranking among the eight South Asian countries are ranking fifth and fourth during the 18 years. exports trade resistance from China to Nepal is larger than bilateral trade resistance; The import trade potential from Nepal to China is huge, the focus of bilateral trade between China and Nepal may be changed, there are more goods may be exported from Nepal to China, and China may become trade deficit when trading with Nepal. Then, the development bottlenecks of the LKSARFT are analyzed. Finally, we give policy directions to boost bilateral trade efficiency and tap the potential of bilateral trade between China and Nepal.

3. The manuscript is not properly organized in terms of (a). Introduction (b). Literature Review (c). Methodology of the study (d). Results and discussion (e). Conclusion and policy Implications.

**Author’s respond**: We have revised the organization of the text.

4. The novelty of the study is not properly outlined. It is recommended to include the novelty of the study at the end of the introduction.

**Author’s respond**: We have added the novelty of the study in the end of the introduction.

The advantages of studying the LKSARFT development prospects by applying SFGM are as follows: (i) SFGM does not suffer from loss of estimation efficiency. (ii) SFGM corrects for the economic distance bias term, which is creating non-normality, isolating it from the statistical error term. (iii) The suggested approach provides potential trade estimates that are closer to frictionless trade estimates, because the approach represents the upper limits of the data, which come from those economies that have liberalized their trade restrictions the most [32]. (iv) The SFGM bears strong theoretical and trade policy implications towards finding ways of minimizing unilateral impacts to volume of trade.

5. It is recommended to include a separate section on “Literature Review”. It is recommended to include in this section a review of previous studies directly or indirectly related to this study. Furthermore, it is recommended to find out the research gap that this study fills.

**Author’s respond**: We have included the separate section on Literature Review.

2.Literature Review

2.1. Bilateral trade potential: based on SFGM

Trade across regions and borders are considered important in improving welfare of people [7,19]. The B&R initiative provides good bilateral development opportunities for both China and Nepal, as well as the other seven South Asian countries. The LKSARFT acts as one of the important land belts, but how about the trade potential of the two countries? What factors promote or limit bilateral trade between the two countries? How to improve bilateral trade efficiency and tap the potential of bilateral trade between the two countries? All these questions’ quantitative study results are helpful to improve the development of the LKSARFT, as well as the development of the B&R. Next these questions are discussed based on stochastic frontier gravity model in detail.

2.2. The stochastic frontier gravity model

Some early literatures have estimated the difference between observed values and the estimated predicted values, by using an augmented gravity equation through Ordinary Least Squares (OLS) to assess the performance of bilateral potential trade among a pair of countries [6,36,15,5]. The OLS estimation procedure produces estimates that represent the centered values of the data set. However, potential trade refers to free trade with no restrictions to trade, some non-efficiency trade factors can’t be observed through this model, so for policy purposes, it is rational to define potential trade as a maximum possible trade that can occur between any two countries, which has liberalized trade restrictions the most, given the determinants of trade. This means that the estimation of the potential trade requires a procedure that represents the upper limits of the data and not the centered values of the data [27]. To solve this problem, [13] proposed the concept of stochastic production frontier analysis which deals with the upper bound of the data set to measure the maximum possible output, this approach is known as the Stochastic Frontier Gravity Model (SFGM). The SFGM is the Integration of Gravity Model and Stochastic Frontier Production Function Model which was formally introduced by [26] to address the inherent bias of the conventional gravity model of trade and to estimate potential trade flows. [53] applied frontier production function analysis to small farms in Nicaragua during 1998-2005, the results showed an acceptable average of technical efficiency which the makers of public policy in Nicaragua a must consider for the future. [40] focused on examining Philippines’s exports efficiency and potential based on trading partner’s characteristics using SFGM, unlike the usual measure of gravity model using OLS that measure potential from the mean. [12] provided a survey about environmental efficiency measure issue. the paper deals with different ways for including environmental variables, which offered several perspectives for measuring efficiency with frontier methods. [45] estimated the potential and efficiency of trade flows between China and countries along the Maritime Silk Road from 2005 to 2013 by using the SFGM. Furthermore, [17] defined the extent of the Belt and Road in terms of geographical boundaries, justified the application of the SFGM to the foreign direct investment (FDI) analysis, and constructed a frontier regression model to assess the China’s outward FDI efficiency. [1] used an extended gravity model to examine the determinants, efficiency and potential of agri-food exports from Nigeria to the EU for the 1995-2019 period the study showed that Nigeria’s agri-food exports with the EU has a relatively large potential that has not been exploited. The stochastic frontier gravity model is applied on a dataset including 35 countries during 2009–2017 by [24], the results indicated that the trade resistance of China’s export to countries along the Belt and Road has increased over time, while there is still huge trade potential at various industries. [2] provided empirical insights on the determinants and potential of agri-food exports from Nigeria to 70 major trading countries between 1995 and 2019 by applying a Stochastic Frontier Analysis on a gravity model. [3] aimed to examine the key determinants and efficiency of China’s agricultural exports with its 114 importing countries by applying the Stochastic Frontier Analysis on an augmented gravity model for the period of 2000–2019.

6. It is recommended that a separate section on “methodology” should include (a). a table regarding “description of the variables” used in this study. (b). It is recommended to discuss the significance of each variable included in this study/model (c). Methods for measuring trading potential between trading partners should be included. (d). It is recommended to include the econometric model that examines the impact of factors including BRI that affect trading potential.

**Author’s respond**: We have added the description of the variables in section 3.

Table 1 The variables

| **Variable** | **Explanation** | **Variable** | **Explanation** |
| --- | --- | --- | --- |
| $\mathrm{EAI}_{ijt}$ | The bilateral trade volume between country$i$ and country$j$ | $\text{EXP}_{ijt}$ | Exports volume from country$i$ to country$j$ |
| $\mathrm{PGDP}_{jt}$ | The per capita gross domestic product in country$i$ | $\mathrm{P}_{it}$ | The total population in country$i$ |
| $\mathrm{PGDP}_{it}$ | The per capita gross domestic product in country$j$ | $\mathrm{P}_{jt}$ | The total population in country$j$ |
| $\text{D}_{ij}$ | The distance between the two countries | $\text{B}_{ij}$ | Situation of border between the two countries |
| $\text{LAN}_{ij}$ | The type of land between the two countries | $\text{LANG}_{ij}$ | Type of language between the two countries |

Also, we have discussed methods for measuring trading potential, please see the discussion in section 4.

The ratio of actual trade volume (EXP and EAI) and efficiency (EE and BTE, presented in Table 6) represents the Exports Trade Potential (ETP) and Bilateral Trade Potential (BTP), respectively. We could obtain the Extended Exports Trade Potential (EETP) and Extended Bilateral Trade Potential (EBTP) by substituting the original data (presented in Table 2 and Table 3) into the estimated random frontier gravity equations. The difference between ETP and EETP (BEP and EBTP) is, ETP (BEP) is influenced by the economic data of other seven countries, and EETP (EBTP) is the economic indicator which reflects the pure economic trade potential between China and Nepal. We subtracted 100% from the ratio of extended trade potential (EETP and EBTP) and trade potential (ETP and BTP), the results presented the Improved Exports Trade Potential (IETP) and Improved Bilateral Trade Potential (IBTP), respectively. All results were presented in Table 7.

Table 7

Trade potential measurement results between China and Nepal (Billion USD)

| **Year** | **Exports trade potential from China to Nepal** | | | | **Bilateral trade potential** | | | |
| --- | --- | --- | --- | --- | --- | --- | --- | --- |
|  | **EXP** | **ETP** | **EETP** | **IETP** | **EAI** | **BTP** | **EBTP** | **IBTP** |
| 2001 | 1.486 | 11.226 | 11.362 | 1.20% | 1.532 | 611.131 | 873.801 | 42.98% |
| 2002 | 1.051 | 14.136 | 18.847 | 33.33% | 1.103 | 556.352 | 759.760 | 36.56% |
| 2003 | 1.220 | 11.590 | 11.146 | -3.83% | 1.273 | 607.900 | 796.939 | 31.10% |
| 2004 | 1.632 | 19.489 | 24.563 | 26.03% | 1.715 | 561.220 | 703.717 | 25.39% |
| 2005 | 1.879 | 18.162 | 16.521 | -9.03% | 1.964 | 621.060 | 743.909 | 19.78% |
| 2006 | 2.598 | 29.419 | 32.576 | 10.73% | 2.680 | 567.938 | 615.128 | 8.31% |
| 2007 | 3.860 | 27.982 | 24.368 | -12.92% | 4.000 | 631.958 | 698.734 | 10.57% |
| 2008 | 3.750 | 54.532 | 61.257 | 12.33% | 3.810 | 586.660 | 622.423 | 6.10% |
| 2009 | 4.090 | 42.652 | 35.940 | -15.74% | 4.140 | 643.052 | 662.756 | 3.06% |
| 2010 | 7.322 | 84.851 | 91.225 | 7.51% | 7.437 | 600.222 | 594.806 | -0.90% |
| 2011 | 11.810 | 89.917 | 72.541 | -19.32% | 11.950 | 667.626 | 638.855 | -4.31% |
| 2012 | 19.680 | 153.796 | 208.593 | 35.63% | 19.980 | 620.408 | 733.035 | 18.15% |
| 2013 | 22.100 | 119.461 | 123.302 | 3.22% | 22.540 | 666.582 | 769.951 | 15.51% |
| 2014 | 22.830 | 150.808 | 200.647 | 33.05% | 23.300 | 617.088 | 693.291 | 12.35% |
| 2015 | 27.890 | 140.424 | 142.450 | 1.44% | 28.660 | 659.878 | 724.848 | 9.85% |
| 2016 | 28.900 | 141.234 | 144.900 | 2.60% | 30.245 | 689.998 | 700.909 | 1.58% |
| 2017 | 31.008 | 150.900 | 157.770 | 4.55% | 32.335 | 699.908 | 711.789 | 1.70% |
| 2018 | 33.450 | 151.990 | 153.890 | 1.25% | 35.667 | 710.990 | 767.897 | 8.00% |

The results in Table 7 show that the BTP is larger than ETP between China and Nepal, also we find that the average IBTP from 2001 is over 15%, but the average IETP from 2001 is just 6%, in 2003, 2005, 2007, 2009 and 2011 the IETP values are negative, which means the import trade potential from Nepal to China is huge, the focus of bilateral trade between China and Nepal may be changed, there are more goods may be exported from Nepal to China, and China may become trade deficit when trading with Nepal. Absolutely, the LKSARFT provides a convenient, safe, time-saving and cost-saving land transport corridor in the trades between China and Nepal.

Since all the data used in this study are collected historical data, which are fixed, and the dimensions of the data are very different, it is troublesome to analyze the importance of variables. Moreover, this study mainly studies the influence of variables on trade potential, rather than analyze variables separately. However, we will discuss the significance of each variable and examine impact of factors that affect trading potential in the future. In response to this, we have added more descriptions of these jobs in the future works part.

Some basic problems, such as operational and management bottlenecks still remained unresolved. How to solve the questions mentioned above, are the further study works; furthermore, distance is the key variable in this paper, and it reflects the transportation cost between two countries and inversely proportional to trade, and the traffic routes and transport modes between China and South Asian Countries are different, so next we will try to improve our work using the distance (between ports, main trading city or capital) multiplied by freight rate (ocean freight, road freight or rail freight) to analyze the bilateral trade between two countries; LKSARFT may reduce the transportation cost between China and Nepal, and even promote trade between China and other South Asian Countries, the new transport lines of LKSARFT are under construction, so next we can also introduce new distance reduced by LKSARFT in the SFGM to predict the trade potential under LKSARFT. Moreover, in order to refine the study, we will discuss the significance of each variable and examine impact of factors that affect trading potential in the future.

7. Referred to Table 1, it is recommended to include descriptive statistics for the data series rather than including the full data.

**Author’s respond**: We have added descriptive statistics for the data series in section 3. Please see the red part.

In this paper we study the LKSARFT, so$\text{D}_{ij}$are the straight line distances between Lanzhou to the capital city of other related countries: Lanzhou-Kathmandu, Lanzhou-New Delhi, Lanzhou-Islamabad, Lanzhou-Dhaka, Lanzhou-Kabul, Lanzhou-Thimphu, Lanzhou-Colombo, Lanzhou-Male.

The results presented in Table 2 demonstrate a steady increase in the bilateral trade volume between China and the eight South Asian countries, as well as China's export volume to these nations from 2001 to 2018. Notably, the bilateral trade volume and export amount between China and India was the highest among the eight countries. The rankings of bilateral trade volume and export amount between China and the South Asian countries from one to eight are as follows: India, Pakistan, Bangladesh, Sri Lanka, Nepal, Afghanistan, Maldives, and Bhutan. Furthermore, the ranking of China's export amount to the eight South Asian countries from one to eight is also as follows: India, Pakistan, Bangladesh, Sri Lanka, Nepal, Afghanistan, Maldives, and Bhutan. The subsequent discussion will primarily focus on Nepal.

8. It is recommended to justify why the author used the SFGM model instead of other econometric models.

**Author’s respond**: We have added the advantages of studying the LKSARFT development prospects by applying SFGM in the end of the introduction.

The advantages of studying the LKSARFT development prospects by applying SFGM are as follows: (i) SFGM does not suffer from loss of estimation efficiency. (ii) SFGM corrects for the economic distance bias term, which is creating non-normality, isolating it from the statistical error term. (iii) The suggested approach provides potential trade estimates that are closer to frictionless trade estimates, because the approach represents the upper limits of the data, which come from those economies that have liberalized their trade restrictions the most [32]. (iv) The SFGM bears strong theoretical and trade policy implications towards finding ways of minimizing unilateral impacts to volume of trade.

9. It is recommended to correlate your study with previous studies conducted. In addition, it is recommended to prescribe the policy implications based on the study results in a separate paragraph after the study conclusions.

**Author’s respond**: We have prescribed the policy implications based on the study results in a separate paragraph after the study conclusions. Please see the Penultimate paragraph.

In line with these findings, we give policy directions to boost bilateral trade efficiency and tap the potential of bilateral trade between the two countries: (i) Export goods would be stimulated by formulating and implementing macro-economic policies aimed at increasing the economic size of China. (ii) The establishment of a robust and comprehensive integrated process for foreign exports would facilitate the process of foreign exports, consequently reducing China's export trade impediments with Nepal. (iii) In order to mitigate transportation costs, China should establish and implement tax incentive policies for cross-border trade in encouraged sectors. (iv) Both countries should promote the teaching of each other's languages in their respective education systems to enhance communication and cooperation, and facilitate cross-cultural understanding, thereby promoting trade development between the two nations. (iv) To rectify China's trade surplus, it is imperative for the nation to calibrate its fiscal policies such that there is a surge in demand, thereby inducing an increase in import volume.

10. It is recommended to enrich the manuscript with a literature review.

**Author’s respond**: We have included the separate section on Literature Review.

2. Literature Review

2.1 Bilateral trade potential: based on SFGM

Trade across regions and borders are considered important in improving welfare of people [7,19]. The B&R initiative provides good bilateral development opportunities for both China and Nepal, as well as the other seven South Asian countries. The LKSARFT acts as one of the important land belts, but how about the trade potential of the two countries? What factors promote or limit bilateral trade between the two countries? How to improve bilateral trade efficiency and tap the potential of bilateral trade between the two countries? All these questions’ quantitative study results are helpful to improve the development of the LKSARFT, as well as the development of the B&R. Next these questions are discussed based on stochastic frontier gravity model in detail.

2.2 The stochastic frontier gravity model

Some early literatures have estimated the difference between observed values and the estimated predicted values, by using an augmented gravity equation through Ordinary Least Squares (OLS) to assess the performance of bilateral potential trade among a pair of countries [6,36,15,5]. The OLS estimation procedure produces estimates that represent the centered values of the data set. However, potential trade refers to free trade with no restrictions to trade, some non-efficiency trade factors can’t be observed through this model, so for policy purposes, it is rational to define potential trade as a maximum possible trade that can occur between any two countries, which has liberalized trade restrictions the most, given the determinants of trade. This means that the estimation of the potential trade requires a procedure that represents the upper limits of the data and not the centered values of the data [27]. To solve this problem, [13] proposed the concept of stochastic production frontier analysis which deals with the upper bound of the data set to measure the maximum possible output, this approach is known as the Stochastic Frontier Gravity Model (SFGM). The SFGM is the Integration of Gravity Model and Stochastic Frontier Production Function Model which was formally introduced by [26] to address the inherent bias of the conventional gravity model of trade and to estimate potential trade flows. [53] applied frontier production function analysis to small farms in Nicaragua during 1998-2005, the results showed an acceptable average of technical efficiency which the makers of public policy in Nicaragua a must consider for the future. [40] focused on examining Philippines’s exports efficiency and potential based on trading partner’s characteristics using SFGM, unlike the usual measure of gravity model using OLS that measure potential from the mean. [12] provided a survey about environmental efficiency measure issue. the paper deals with different ways for including environmental variables, which offered several perspectives for measuring efficiency with frontier methods. [45] estimated the potential and efficiency of trade flows between China and countries along the Maritime Silk Road from 2005 to 2013 by using the SFGM. Furthermore, [17] defined the extent of the Belt and Road in terms of geographical boundaries, justified the application of the SFGM to the foreign direct investment (FDI) analysis, and constructed a frontier regression model to assess the China’s outward FDI efficiency. [1] used an extended gravity model to examine the determinants, efficiency and potential of agri-food exports from Nigeria to the EU for the 1995-2019 period the study showed that Nigeria’s agri-food exports with the EU has a relatively large potential that has not been exploited. The stochastic frontier gravity model is applied on a dataset including 35 countries during 2009–2017 by [24], the results indicated that the trade resistance of China’s export to countries along the Belt and Road has increased over time, while there is still huge trade potential at various industries. [2] provided empirical insights on the determinants and potential of agri-food exports from Nigeria to 70 major trading countries between 1995 and 2019 by applying a Stochastic Frontier Analysis on a gravity model. [3] aimed to examine the key determinants and efficiency of China’s agricultural exports with its 114 importing countries by applying the Stochastic Frontier Analysis on an augmented gravity model for the period of 2000–2019.

**We sincerely thank all reviewers and editors for your valuable advices and comments on this paper, because the advices and comments are great helpful to improve this article. If there are any other problems and questions need to be handled in this paper, please tell us, thank you so much!**
